# Morphia in Cholera Infantum

**Published:** 1885-08

**Authors:** 


### Morphia in Cholera Infantum.

Dr. Spencer M. Free, of Pennsylvania, writes in the *American Journal of Obstetrics*, claiming that true cholera infantum (not the other bowel difficulties, improperly called cholera infantum) is so similar to sporadic cholera of adults, commonly known as cholera morbus, that the same principle of treatment was suggested to his mind.

He reports cases occurring in children from one to two years old, in which he gave morphia, either hypodermically or dry upon the tongue, followed by the same prompt and beneficial effects observed after the administration of morphia in the disease in adults, namely: sleep, rest from vomiting, and diarrhœa. "Nature is afforded an opportunity to recover her balance." He insists that the dose must be large enough to produce full physiological effects, and it must be given, hypodermically or dry, on the tongue, otherwise the little patient will vomit it, if given in solution.

He has given as high as one-twelfth grains dry on the tongue to a child fifteen months old.

